# Mechanized Significance and Machine Learning: Why It Became Thinkable and Preferable to Teach Machines to Judge the World

**DOI:** 10.1007/978-3-030-56286-1_2

**Published:** 2020-12-01

**Authors:** Aaron Mendon-Plasek

**Affiliations:** 1 Centre Urbanisation Culture Société, Institut national de la recherche scientifique, Quebec City, QC Canada; 2 Centre for Interdisciplinary Methodologies, University of Warwick, Coventry, UK; Department of History, Columbia University, New York, NY United States

## Abstract

The slow and uneven forging of a novel constellation of practices, concerns, and values that became machine learning occurred in 1950s and 1960s pattern recognition research through attempts to mechanize *contextual significance* that involved building “learning machines” that imitated human judgment by learning from examples. By the 1960s two crises emerged: the first was an inability to evaluate, compare, and judge different pattern recognition systems; the second was an inability to articulate what made pattern recognition constitute a distinct discipline. The resolution of both crises through the problem-framing strategies of *supervised* and *unsupervised learning* and the incorporation of statistical decision theory changed what it meant to provide an adequate description of the world even as it caused researchers to reimagine their own scientific self-identities.

Wherever and whenever groups of people come to agree about what knowledge is, they have practically and provisionally solved the problem of how to array and order themselves. To have knowledge is to belong to some sort of ordered life; to have some sort of ordered life is to have shared knowledge.^1^

— Steven Shapin and Simon Shaffer, *Leviathan and the Air*-*Pump*

## Judging Learning Machines and Making Comments Toxic

Machine learning is often seen as either an early-twenty-first or late-twentieth-century subfield of artificial intelligence drawing on techniques in the fields of operations research, cybernetics, cognitive science, and statistics.^2^ This chapter, by contrast, shows how the problem-framing strategies and practices of machine learning now in ascendancy were articulated, made durable, and widely circulated by researchers working on pattern recognition problems from the 1950s to the 1970s.^3^ Developed through the interactions between different communities, research problems, and computing devices in 1950s and 1960s, pattern recognition researchers sought to mechanize the identification of contextual significance, standardize comparisons of different machine learning systems, and codify a body of training, techniques, and reasoning by inaugurating a new discipline. The slow and uneven forging of a novel constellation of practices, concerns, and values through pattern recognition research changed both what it meant to provide an adequate description of the world even as it caused researchers to reimagine their own scientific self-identities.

Early mid-1950s pattern recognition researchers articulated a conception of *contextual significance* that could be performed by humans or machines, and that emphasized performance *reliability* in parity with human judgment and *robust* performance in the face of never-seen-before new data. This notion of *contextual significance*, as discussed in section “How Identifying Significance Became a Branch of Engineering”, was exemplified and circulated in the Cold War research problem of optical character recognition (henceforth *OCR*), and encouraged a form of problem solving that sought to produce useful decisions purely through learning from examples. The multiple meanings of *learning* in pattern recognition, identified in section “Pattern Recognition Eats the World: Learning as a Solution to Technical Problems and to Questions of Social Order”, were aspirational in that they were expressions of researcher ambitions for what machines *could* and *should* be taught to do. This polysemous *learning*, instantiated mathematically in late 1950s pattern recognition as a *loss function* borrowed from statistical decision theory, fused day-to-day technical decisions for building pattern recognition systems with attitudes that linked *creativity* to mechanical schemes for generating contextual significance. The theoretical conception of “learning” from examples offered as a solution to the problem of contextual significance was analogically seen as a solution for deciding social questions. This occurred, as sketched out in section  “Incomparable Alphabet Learning Machines and a Game with the World”, because of two questions of knowledge that pattern recognition researchers faced in the late 1950s and 1960s. The first question was at the level of the individual laboratory: namely, how to evaluate, compare, and judge different OCR systems which used different methods, devices, and data sets? Establishing learning criteria also had a pragmatic benefit of having the pattern recognition systems judge and improve their own performance given new data. The second question was one of professional identity: namely, what made pattern recognition constitute a distinct discipline deserving of its own conferences, jobs, and funding? Both questions tied workaday technical practices to national funding agencies and transnational communities of knowledge production; but, more importantly, both questions were interwoven with each other such that the forms of learning offered as a solution to one question at the level of individual labs constrained disciplinary possibility at the national level, and vice versa. Researchers found a solution to these duel questions of how to judge pattern recognition systems and how to legitimize their discipline via the mid-1960s problem-solving strategies of *supervised* and *unsupervised learning*.

Researchers seeking to make pattern recognition into a reputable field of inquiry saw their object of study as the mechanical identification of significance and the reproduction of human judgment. They emphasized building “learning machines” that reliably performed a specific task within a narrow context despite wide variations in input, and were tied to a way of formulating problems that encompassed disparate activities: spotting missiles in the sky or spotting card catalog subjects in thousands of papers; disambiguating polysemy in machine translation or disambiguating particle tracks in bubble chambers; identifying unstable governments in the Cold War world or identifying individual alphanumeric characters on paper. The contextual flexibility and robustness of pattern recognition was tied, in part, to letting the phenomenon of interest be defined by the statistical properties of the data. “Solutions to the problem of knowledge,” Shapin and Shaffer have compellingly argued, “are solutions to the problem of social order.”^4^ For theoretically-inclined pattern recognition researchers there was little difference between looking for patterns in the natural world and looking for patterns in human society. If humans and machines could make the same classifications in a particular task (e.g., to recognize the letter “A,” a tank, a fingerprint, a population), machines could make social judgments.^5^ Because *significance* in pattern recognition was tied to a way of problem-framing for which *learning* was seen as the answer, mere narratives of the development of supervised and unsupervised learning miss the point. Textbook definitions of supervised and unsupervised learning as self-evident synchronic ordering principles of machine learning miss the point: for the historian it is precisely *why* these forms of learning became stable, durable ordering principles that *need to be explained.*^6^ To trace the shifting notion of *learning* in the larger matrix of tacit problem-framing strategies shared by pattern recognition researchers, we need to look at “substitut[ing actions] for concepts and practices for meanings.”^7^

Examples from the history of science are instructive. To historicize scientific objectivity, Daston and Galison (2010) adopt a “bottom-up” historiographic method in which “ideal and ethos are gradually built up and bodied out by thousands of concrete actions, as a mosaic takes shape from thousands of tiny fragments of glass.”^8^ To follow the changing “collective” knowledge and the various kinds of disciplined “knowing sel[ves]” that such collective knowledge requires, they examine the images of scientific atlases and the practices and values these atlases manifest. I sketch a genealogy of *learning* in the then-nascent field of pattern recognition in the 1950s and 1960s, including how technical and professional contingency informed practices of communal- and self-valuing, by a careful examination of the central research problem of pattern recognition at that time: optical character recognition. OCR research in the 1950s and 1960s did not center on one algorithm or one broad collection of techniques, but articulated a set of specific concerns built into the pattern recognition problem-framing for which messy data was no more or less preferable to high-quality, expensively produced, well-curated data sets. While OCR work did not provide prestige and visibility to the field of pattern recognition, the development and circulation of pattern recognition work in the 1960s across a variety of domains, and its attendant forms of *learning*, provided a blueprint for *how* to solve a problem and, more importantly, what *counted* as a good problem.

Today machine learning reformulates the original sin of incomplete knowledge into an epistemic virtue of greater contextual sensitivity. For example, Google’s *Perspective* API uses a machine learning classifier to estimate the likelihood of a comment to be “perceived as ‘toxic,’” and was intended to “help increase participation, quality, and empathy in online conversation at scale” by curtailing “abuse and harassment online.”^9^ The classifier was trained on comments labeled “toxic” or not, but to have enough labeled comments a data set first had to be produced. Crowd-sourced workers rated more than 100,000 Wikipedia talk page comments from “very healthy” to “very toxic” according to how they saw each comment as “a rude, disrespectful, or unreasonable comment that is likely to make you leave a discussion.”^10^ The ambiguity of the definition was purposeful, in the hopes of facilitating decision-making more responsive to the contextual and positional ambiguities spanning race, gender, nationality, ethnicity, sexuality, and class in disparate “toxic” commenting practices. As Perspective’s project manager has said, “We want to build machine-assisted moderation tools to flag things for a human to review, but we don’t want some central definition or someone to say what is good and bad.”^11^ A neural network classifier was then trained using the comments and human classifications to replicate the human ratings for each comment.^12^ This classifier, so trained, was used “as a surrogate for crowd workers” to label all *63 million* Wikipedia talk page comments spanning 2004–2015 as “toxic” or not.^13^ In effect, to both those being surveyed and Perspective’s developers, what constitutes “toxicity” is known by its imagined effect, and only ever probabilistically. While such *ignorance*-*as*-*generality* may rightly give us pause when applied to high-stakes decisions, the advantages of this strategy in low-stakes decisions become more apparent when applied to the problem of OCR discussed in the next section.

We can see other problem-framing concerns endemic to machine learning in the case of Perspective, especially in how machine learning systems are fixed when discovered to make improper decisions or classifications. With the *New York Times*, the *Economist,* Wikipedia, and the *Guardian* emblazoned on Perspective’s website as “partner experiments,” Perspective’s public release in 2017 by Google and Jigsaw soon exposed that Perspective’s classifier rated comments with words denoting historically marginalized gender, sexual, and racial identities (e.g., “woman,” “black,” “gay,” “asian,” “homosexual,” etc.) as more probable to be perceived as toxic.^14^ The classifier’s problem, developers contended, was that in the training corpus these identity words were “over-represented in abusive and toxic comments” because these terms disproportionately appeared in online attacks.^15^ The “toxicity” classifier trained on this data “overgeneralized and learned to disproportionately associate those [identity] terms with the toxicity label.”^16^ The solution, developers continued, was to de-toxify these identity words in the corpus by adding more nontoxic comments containing identity terms and then retraining the classifier on this new corpus. Fixing the classifier by adding more data under the justification of bringing the training data into greater fidelity with social reality *did* achieve the desired technical result, and, for our purposes, illustrated two key epistemic values. First, for Perspective’s “toxicity” classifier to work, it needs to be able to identity *contextual significance*: namely, that the same data (e.g., words, n-grams) may produce different effects (e.g., a person leaving a conversation or staying) in different contexts (e.g., different online platforms, different identities). Second, in order to be useful even when encountering new forms of toxic speech, the classifier needs *contextual robustness:* namely, that decisions or categories (e.g., “toxic” speech) developed from past data (e.g., the training corpus) will continue to be correct for future as-yet-unseen data. As we shall see in the following section, *contextual significance*, *contextual robustness*, and *ignorance*-*as*-*generality* were carefully constructed epistemic hedges tied together through a conception of *mechanized significance* in mid-century pattern recognition problems.

## How Identifying Significance Became a Branch of Engineering

“ Pattern recognition,” Oliver Selfridge wrote for a 1955 Western Joint Computer Conference session on “Learning Machines,” is “the extraction of the significant features … from a background of irrelevant detail” performed by a machine that “learns” by “build[ing] up sequences [of mathematical operations] ‘like’” but not identical to those previously efficacious at a specific task.^17^ The chair of the session, Willis Ware, then at RAND, explicitly noted the “‘Giant Brain’ ballyhoo” surrounding “learning machines” and observed that these programs “may be said to ‘think’ only to the extent that their users and designers have been able to penetrate sufficiently deeply into a problem so that all possible situations which might arise have been examined[.]”^18^ This machine learning was not learning to accommodate new tasks in completely unforeseen situations.^19^ Nor was this the learning of Allen Newell’s “ultracomplicated problems” that “deal[t] with a complex task by adaptation” in which “relevant information is inexhaustible,” “potential solutions [are] neither enumerable nor simply representable,” and that took chess as the exemplar problem par excellence.^20^ Rather, the open-ended research question, as Selfridge’s collaborator Gerald Paul Dinneen put it, was

to design a machine which will recognize patterns. We shall call any possible visual image, no matter how cluttered or indistinct, a configuration. A pattern is an equivalence class consisting of all those configurations which cause the same output in the machine.^21^

“Good” features were obtained by mathematical “sequences of operations” performed on the digitized images whose values empirically distinguished between equivalence classes for the letters *A* and *O*.^22^^,^^23^ Such good features were ideally insensitive to “variations [that] ought not affect the valuation” like letter size, position, angle, and letter font.^24^

Rejecting straight lines as “too simple” a task and faces as “too complicated,” Selfridge and Dinneen settled on identifying printed black “A”s and “O”s on paper, which were chosen for their respective asymmetry and symmetry.^25^ In contrast to AI and its operations research and proof-proving roots, the point was not “to build an efficient reading machine, but rather to study how such a machine might work” to handle the enormous variability of input letters—especially *A*s and *O*s the machine had never encountered and even beyond what the Lincoln Lab researchers could anticipate.^26^ This situation was *not* the same as AI’s mid-1950s obsessions with heuristic problem solving of mathematical questions, automatic translations facilitated by programming compilers, or logistical operations of bureaucracies. Pattern recognition for Selfridge and Dinneen was about mechanizing a particular notion of contextual significance that valorized the reliability of machine performance (i.e., correct classification) for a task given novel data with deformations that are not known or anticipated at the outset.^27^

Such contextual significance meant that the letter *A* might differ for two different sets of “A”-labeled images in potentially contradictory ways. This might appear to be a wildly idiosyncratic way of identifying anything—whether letters, EKGs, faces, fingerprints, or cats—hopelessly sensitive to the peculiarities of the features selected and the training data used. However, Selfridge reframed this sensitivity to individual examples as a virtue. By identifying a problem in which such a strategy was preferable—namely, that of *optical character recognition*—and noting analogically how this sensitivity to training data was what facilitated humans’ comparably superior capacity for finding significance, Selfridge made experience the sole source of contextual significance:

Now significance is a function of, first, context, and second, experience…. [O]f course, context is a function of experience. But more than that, *experience alone affects the kind of thing we regard as significant.*^28^

If humans could learn significance (and indeed, everything else that they can learn) solely through experiencing the world, Selfridge contended, why couldn’t machines learn an image recognition task solely from labeled images? The “recognition of simple shapes in a visual field, such as block capital letters,” was a useful test case.^29^

But how did identifying two letters generalize in any way to a method for identifying contextual significance? As seen above, for Selfridge and Dinneen the letters *A* and *O* each constituted an *equivalence class*, which was defined as whatever feature values consistently picked out “A”-labeled images as *A*s and “O”-labeled images as *O*s.^30^ These equivalence classes were not an ideal *A* or *O* around which each letter image was an imperfect copy. Rather, an equivalence class defined the values of a feature (or features) that categorized images as the same letter: say, many different “A”s as *the* letter *A*, even if all “A” images have “no one thing in common that makes us use the same [letter name] for all.”^31^ This is why equivalence classes were so named: pattern recognition was, Selfridge contended, “classifying configurations of data into classes of equivalent significance so that very many different configurations all belong in the same equivalence class.”^32^

This method illustrated what researchers would later call the “two subsidiary problems” of pattern recognition: “what to measure and how to process the measurements.”^33^ Selfridge’s answer to the former problem dictated his answer to the latter, forming a hermeneutic circle between observed image *features* (i.e., the series of preprocessing operations performed on each letter), the data *configurations* (i.e., the collection of labeled “A” and “O” images), and *equivalence classes* (i.e., the particular feature attributes that distinguish *A*s and *O*s). Building, evaluating, and improving a learning machine could only be achieved by understanding the relations between individual *features, configurations, and equivalence classes* themselves and the machine’s performance as a coherent whole in relation to these individual parts. More or less useful features and equivalence classes could be determined via iterative computational guessing and checking different combinations of *features, configurations,* and *equivalence classes*. Any “sequence of operations” that produced features with values that distinguished *A*s and *O*s could work and could be checked via (potentially laborious) trial and error—whether digital computers or humans were doing the trial and error, for Selfridge, mattered not.^34^

Learning as feature selection improvement in pattern recognition did not foreground expertise, in part, because, as Ware noted, Selfridge’s and Dinneen’s learning machine papers dealt with “the learning of pattern recognition by a maturing individual” (i.e., a child learning to read) while Newell’s problem of chess-playing expertise examined “the action of an entire man as he copes with complicated problems of his life.”^35^ Ware’s pronouncements had the form of normative statements about task complexity, but, in practice, were a statement on the permissible simplifying assumptions for problem inputs precisely because this was one strategy for comparing one problem to another. For Selfridge and Dinneen, input letter images were “well organized in two spatial dimensions and exhibit[ed] a well defined topology”; for Newell, problem inputs were “the highest level of complexity—[that of] the game” and involved “a multitude of input data which must be inspected at many levels.”^36^ Both Selfridge and Dinneen’s OCR problem and Newell’s chess-playing program had to learn “criteria for decision[s that] are a priori unstated.”^37^ Already between the letter-learning and chess-playing problems were all the hallmarks of what Newell would later identify as two distinct dispositions “to construct [scientific] theories” dictating for the researchers “what is important, what problems can be solved, [and] what possibilities exist for theoretical extension,” including the ways in which learning criteria were specified.^38^ Pattern recognition researchers, like Selfridge and Dinneen and others at the Lincoln Laboratory, employed “sets of continuous variables as the underlying state descriptions,” and were trained in the problems and problem-solving strategies of electrical engineering.^39^ So informed by training and the task of building an OCR machine, pattern recognition, as exemplified by Selfridge’s and Dinneen’s 1955 papers, offered a vision of a nascent disciplinary formation that combined different notions of *learning* that reflected these researchers’ ambitions for a machine able to distinguish *contextual significance* while maintaining *contextually robust* performance when encountering new data.

## Pattern Recognition Eats the World: Learning as a Solution to Technical Problems and to Questions of Social Order

What could people do using pattern recognition that they couldn’t do before? What made pattern recognition’s problem-framing not merely rhetorically compelling but intellectually preferable for some communities? Which communities celebrated and were empowered by these capacities? Pattern-learning machines offered a way of imperfectly knowing the world via its provisional and piecemeal traces.^40^ Mid-century pattern recognition shared with mid-century cybernetics what Andrew Pickering calls a “black box ontology,” in which the world is filled with black boxes “that [do] something, that one does something to, and that does something back,” and for which its internal workings are opaque to us.^41^ Pattern recognition systems, like the cybernetic systems Pickering discusses, attempted to “go on in a constructive and creative fashion in a world of exceedingly complex systems” that might never reasonably be understood or derived from first principles.^42^ Given these parallels between cybernetics and pattern recognition, it is no surprise that Selfridge worked as Norbert Wiener’s assistant in graduate school, and copyedited a draft of Wiener’s *Cybernetics.*^43^ The framing of Selfridge’s 1955 pattern recognition paper echoes cybernetics’ “ontology of unknowability” in how, for example, the ideal letter *A* is never known or pregiven, but an effective equivalence class can always be learned. For all their similarities, however, pattern recognition distinguished itself from cybernetics in valorizing knowledge as a kind of classification. Representing phenomena as aggregates-of-features was a strategy for reproducing (human) judgment (insofar as these “judgments” could be translated into classification decisions) that also held the possibility of being robust to unexpected new data.

Researchers working on pattern recognition problems were engaged in an intellectually stimulating but professionally fraught endeavor in the 1950s and 1960s since pattern recognition was seen by many as applied engineering and not an intellectual arena able to facilitate professional advancement. Work in pattern recognition was accordingly often conducted as a tertiary research pursuit. Selfridge, Dinneen, and others did pattern recognition research informally, during lunch hours.^44^ Their ability to use the Test Memory Computer at Lincoln Laboratory to conduct machine learning experiments was made possible, in part, by the computer’s reduced use as a support for the Whirlwind Computer employed in the service of the Department of Defense’s Semi-Automatic Ground Environment (SAGE) project.^45^ Established disciplines’ funding for pattern recognition projects was often in service to a particular military problem to be solved in corporate labs like Bell, Philco, IBM, RAND, and GE, and were in sharp contrast to the few-strings-attached ARPA funding of coeval AI research found in university labs at MIT, Carnegie Mellon, and Stanford often discussed in the history of computing.^46^

Those working on pattern recognition and seeking professional advancement through the pursuit of a disciplinary identity were confronted by pattern recognition’s methodological disunity and a vast sea of clever, ad hoc mechanical jury-rigged prototypes. The strategies that developed in response to the problems of recognizing alphanumeric letters in particular, both typed and handwritten, on paper and elsewhere, was a key site in which virtues were formed and justified. Pattern recognition work, especially OCR work in laboratories, in panels at international conferences, government funding agencies, and in individual lives was a critical trading zone that shaped these concerns about how to know the world. What was shared by early pattern recognition research was a set of intellectual ambitions, problem-framing strategies, institutional priorities and norms, and computing practices endemic to OCR work exemplified by Selfridge and Dinneen’s 1955 efforts. OCR facilitated the circulation of pattern recognition’s methods and values because of its practical application in a variety of disparate domains as well as for the ease with which its methods were generalized and repurposed for non-OCR problems.^47^

A key facet of the “black box ontology” in pattern recognition’s framing of good problems was the plurality of meanings of *learning*. In practice, these different meanings of learning were expressed mathematically by the use of the *loss function* that represented the numerically-defined “costs” of a particular decision or action. While pattern recognition learning had not yet become synonymous with minimizing the loss function by the early 1960s, this form of learning was seen as a viable strategy to realizing three ambitions. First, this conception of learning could serve as a decision procedure for *mimicking* (human) judgment for narrow, well-defined cases. Second, this learning *generated* trust through its repeated use when, as researchers described it, “we do not understand our problem well enough to pre-program reasonable sets of characterizers.”^48^ Third, the idea of learning as “problems of successive estimation of unknown parameters” served as an aid to solving problems when the optimal method was unclear but data was plentiful.^49^ All three notions of learning were aspirational, and all three constrained and spurred local day-to-day engineering decisions about how to build, compare, and improve pattern recognition machines, and what forms of professional advancement were available to researchers. More importantly, these conceptions of learning informed what constituted knowledge and the human capacity for identifying significance, and continue to buoy and justify claims of machine learning efficacy today. We treat each of these three in turn.

### Mechanical Schemes for Imitating Human Judgment

Pattern recognition provided a strategy for reproducing *any* classification humans could make for a finite collection of images (or any other categorical data) *by definition*. Insofar as classification was a synecdoche for significance, identifying importance became a problem of engineering. Finding the image features correlated to whatever humans employed in the same classification was a problem that could itself be partially automated. Picking out *A*s and *O*s became possible not only for labeled images (where you already had the answer), but also for *A*s and *O*s in unexpected images not anticipated by programmers themselves. This strategy could be applied to a universe of decision problems, as was articulated by pattern recognition researchers by the late 1960s:

[W]e often speak of the character-recognition problem, the problem of analyzing electrocardiograms, the photointerpretation problem, and so on. Yet each of these is in reality not a single problem but a problem area, an entire ensemble of problems. The problems within an area are generally governed by a number of important parameters. Each proposed application, each choice of parameters, specifies a new problem from the ensemble—there are literally an infinity of problems.^50^

This ambition to produce a methodology able to handle any and all decision problems led to a disciplinary identity crisis for pattern recognition similar to that experienced by statistics in the twentieth century and data science today, and which manifests in the confusion and conflation of the terms artificial intelligence and machine learning.^51^ That pattern recognition might be a generalized classification procedure was a self-evident good to be much lauded. But the ambition of universal applicability for many pattern recognition researchers as a completely general form of machine learning also contributed to much hand-wringing in the 1960s and early 1970s about what made pattern recognition a unique field distinct from other engineering or scientific disciplines.

### Making Computers a Reliable Interface with the World Through Patterns-for-Agents

 Pattern recognition was a hodgepodge of technical and social strategies for inventing and ensuring *reliability* for machines directly interfacing with a messy, inconsistent, and ever-changing real world. What reliability was, or, how it could be measured, often came down to local laboratory decisions about what constituted a “task” and how that task was to be integrated into the sensibilities, infrastructures, and worldview of those needing the task to be done.^52^ The decision to restrict pattern recognition tasks to an extremely narrow range of actions and possibilities for purposes of reliability also became a strategy to establish credibility. Pattern recognition, Donald MacKay wrote, “cannot ultimately be separated from that of the organization of action: activity in view of ends.”^53^ In stark contrast to some applications of machine learning today, the fact that mid-century pattern recognition was *limited* to a specific context of use for a particular agent was considered a *virtue* of the approach. Pattern recognition implicitly had “the notion of pattern-for-an-agent” built in such that “the problem of pattern-recognition is always ill-defined until the class of agents in question, and their repertoire, have been specified in relevant detail.”^54^ The kind of trust provided by patterns-for-an-agent arose through the machine’s repeated use in well-defined situations and not from computing operations per second, the speed and capacity of computer memory, or the availability of ever-larger digitized data sets.

Today we are sensitive to how the repurposing of an existing pattern-for-an-agent can produce fictitious inductive claims, and that when such pattern learning machines are used to arbitrate or assist in the adjudication of social decisions involving historically marginalized groups, such reused “patterns” produce unjust, unfair, and inegalitarian decisions. Selfridge’s articulation of pattern recognition and its implementation by Lincoln Lab researchers may strike contemporary readers as woefully unconcerned about the idiosyncrasies of their labeled training data.^55^ Such concerns, though justified, erase the intellectual virtues that produced and constrained the possibilities for a particular system to establish credibility.^56^ Curation of training data for a particular use case was provisional, as we have seen with Selfridge and Dinneen’s 1955 work, in that it relied on how features changed across available but arbitrary examples: as MacKay put it, “our notion of pattern is relative, [but] it is not on that account subjective.”^57^ What made classifications stick was relative difference: there was no absolute definition of classification to which one would *want* to appeal. Nor, for the problems like OCR or image classification, was there a special set of data to *appeal to* because the techniques were a strategy for machine performance reliability.^58^ What they did have to identify were the variability of important differences to the problem. However, picking features, as just one decision of many in building a pattern recognition system, tended to be based “upon intuition, personal feelings, and preferences,” a host of pragmatic engineering concerns, and the particular system in which it would be used.^59^

For pattern recognition researchers, a particular learning machine gained its credibility not through mathematical proof but by its consistent uniform performance at a specific task that was responsive to and not incapacitated by unexpected, even contradictory, information. Researchers building learning machines funded by the military and employed in university labs and in commercial corporations didn’t need a machine to generate its own goals in a well-defined axiomatic system as was espoused by some prominent mid-century researchers in artificial intelligence.^60^ Pattern recognition researchers instead needed a machine to reliably perform a well-defined task in which the enumeration of all potential inputs or the rules governing these inputs was not pragmatically possible.^61^ By 1961 OCR systems still required a “consistently high quality of the input patterns, and [had] a limitation of the recognizable alphabet to at most several hundred members *with each allowable size and style variation of each character counting as a separate member*.”^62^ Reliability was often as much of an aspiration as it was an established fact of practice. This placed concerted attention in pattern recognition on the “prerecognition image processing,” to clean up the image, and which, in fact, was inherently “dangerous” since removing the “salt and pepper” imperfections on character images “without first recognizing the character … will undoubtedly do harm at times, and do some good at other times.”^63^

### A Decision Procedure When You Don’t Know the Problem Solution Space

Insofar as pattern recognition had a methodological theory, it was through an “effective transformation of a perception-recognition problem,” such as optical character recognition,“into a classification problem.”^64^ Such a transformation informed the ways in which data was to be valued, and how the identification of significant information might be *performed and judged*. Which statistical techniques were brought to bear in a particular pattern recognition project varied, in part, on whether the “pattern” in question was for use in (1) textual work (e.g., OCR, information processing, machine translation, hand-printed letters, etc.), (2) social and scientific judgment, data processing, and surveillance (e.g., bacteria colony formation, emission line spectra, tank images, maintenance schedules, fingerprinting, speech processing, etc.), or (3) the studies of “ perception,” including efforts to build and employ neural networks.^65^ Of these three, from the late 1950s to early 1970s, OCR circulated throughout the pattern recognition community as a key exemplar problem from which practitioners learned, remixed, and reified problem-solving strategies, and that brought together practitioners with different skills, training, and goals.^66^ OCR work facilitated the introduction and circulation of statistical decision theory as a particular method and form of solution that reshaped the ambitions of pattern recognition, as one pattern recognition researcher wrote in 1968, from a “narrow esoteric field of electronic computer applications” to “one of the most ambitious scientific ventures of this century[.]”^67^ The following section investigates how this transformation of pattern recognition from “esoteric” to universal occurred through a sustained inability of researchers to agree upon the appropriate ways to measure machine learning performance in OCR pattern recognition systems. The use of decision theory’s loss functions was one strategy that fostered this transformation. The loss function was made durable, in part, by the loss function’s ability to satisfy social, political, and disciplinary needs as well as facilitating the intellectual creativity of both statisticians and engineers involved.

## Incomparable Alphabet Learning Machines and a Game with the World

The overwhelming predominance of OCR systems as the de facto use case of pattern recognition ensured that the process of accounting for variance in training “features” was often decisive to a system’s effectiveness. In practice, as late as 1968, commercial OCR systems compared test characters against ideal character “masks” for typed text, which, while a technique of pattern recognition, was not an example in the machine learning tradition that this chapter has been tracking.^68^ Identifying characters via a set of features was sometimes used to identify handwriting, but such strategies tended to produce more errors as a function of the number of possible characters and were usually more computationally intensive than mask approaches.^69^

The practice of learning typed letters and typed letter features empirically from training data as outlined by Selfridge in 1955 remained more of an aspiration in the early 1960s than a physically realized working system, but this may have reflected a belief about the demand for “self-programming reading machines.”^70^ “No one,” writes J. Rabinow in 1968, “has ever asked us to build [a self-programming reading machine]—not even semi-seriously.” Rabinow continues:

Most of the reading machines built today are used in business where the OCR machine reads a prescribed font. In most other situations the number of fonts is quite limited and 5 to 10 specific fonts are usually more than enough. In the case of the Post Office, particularly in the case of zip codes, we can normalize the size and do some feature analysis and guess the numbers. But if one wanted to build a self-programming alphanumeric reader for the Post Office, one would be faced with the fact that there just isn’t enough information. This is true both because the number of characters to be read on any envelope is too small, and because one envelope has no relationship to the one before…. As far as I know, there is no self-programming machine, but we all think it would be fun to make one.^71^

Yet there were already considerable efforts underway to identify hand-printed alphanumeric characters in the same conference proceedings containing Rabinow’s statement. An ARPA funded project at RAND called GRAIL tracked a user’s light pen strokes to correctly recognize character inputs “with about” (Groner’s words) 90–95% accuracy largely for the purpose of making programming flowcharts.^72^ The System Development Corporation, also supported by ARPA, built a similar light pen system that did the same thing with no recognition accuracy mentioned.^73^ Both used dictionaries of pen stroke gestures associated with each symbol.

 An OCR problem similar to Selfridge’s was pursued by John Munson at Stanford Research Institute (SRI) with funding from the US Army and Navy. Here the task was to take paper “Fortran coding sheets” at the Applied Physics Laboratory at SRI, filled out by hand, and accept them as Fortran programs to be run directly by the machine after being scanned. First, the SRI team used a TV camera to ingest the handwriting characters as 24 by 24 black-and-white grids for “3 alphabets of FORTRAN characters from each of 12 writers” from which character features were calculated with “three 85-weight dot product units per category[.]”^74^ Alphabet sets from four additional writers were used to test the system’s capacity to recognize Fortran characters, which “achieved a correct classification rate of 80%” but for which Munson estimated could be “raised to 85%, or at best to perhaps 90%.”^75^ Their future work entailed building an “ad hoc” method using “a decision tree, a huge look up table, a weighted decision, and adaptive process, or a combination” that would provide a list of answers accompanied by confidence probabilities that would achieve “on the order of 90% or better” for individual characters.^76^ For the specific task of reading Fortran code as input, they hoped to use Fortran syntax itself to further correct letter recognition so as to “produce text of 99% accuracy or better.”^77^

Despite such optimism, Munson was painfully aware that such error rate measures were themselves highly idiosyncratic, depending on the way the data set was created, digitized, and stored, the various learning procedures employed, the kind of classification process attempted, and the criteria for success. This made it dubious to attempt to compare reported error rates between different systems or OCR projects. Munson concluded:

We view the problem [of handprinted text recognition] in the five stages of data preparation, data input, preprocessing, classification, and context analysis…. We believe that these stages must all be kept in view, however, because a change in performance in one greatly affects the demands put on another. We have tried to establish a framework in which, if the outcome of an experiment is x% correct classification of characters or lines of text, the meaning and value of the result will be clear.^78^

The prevalence of a prototype-and-try attitude toward pattern recognition systems by engineers further exacerbated the difficulty of comparing the efficacy of different OCR programs across different data sets. An OCR program that worked well for one data set would do poorly for a different data set. The issue was not simply what contemporaries might call “overfitting,” but rather that the data sets might have antithetical feature values for the same equivalence class.^79^ A researcher could always select an ad hoc criteria to compare different machine learning systems, but such comparisons required much labor, money, and institutional infrastructure. Given that most projects were task specific, few resources were usually given for building standards across projects, organizations, or institutions, except when demands were imposed by research funders. Any claims about the “correctness” or “generalizability” of learning programs to “read” the zip codes for the US Post Office,^80^ the finance forms for the US Army, student information for the Chicago Board of Education, or driver’s license applications for the State of Michigan, or for “automatic newspaper [re]production” for international publishers, were entirely pegged to their institutional contexts and assumptions, and rendered incommensurable when these “tasks” were considered outside of the insitutions in which they were performed.^81^ In practice, other concerns could render pattern recognition system accuracy comparisons moot: cost-effectiveness, for instance, was often prioritized even over higher recognition accuracy.^82^

Theoretical studies of pattern recognition were grounded in statistical decision theory as well as signal-to-noise problems in radar, telecommunications, and other applications of information-theoretic measurements. Building on both Fisher’s and Pearson and Neyman’s hypothesis-testing frameworks, statistical decision theory involved calculating a problem-specific “decision function” to decide how many “stages” of experimental observations were necessary before making a “terminal decision” about which hypothesis to accept given the opportunity cost of incorrect decisions and the possibility of additional experimentation.^83^ Much of the initial development of decision theory was done by Abraham Wald, who fled Nazi-occupied Austria to the US in 1938 via the assistance of the Cowles Commission, joined Columbia University’s faculty in 1941, and would die in a plane crash in India in December 1950.^84^ During this brief time, Wald’s work made important contributions to statistics: first, by his singular explication of statistical decision theory in 1939, and, second, via his development of sequential analysis to decision theory in 1943 as part of the Statistics Research Group at Columbia.^85^ Both efforts were combined in his definitive Office-of-Naval-Research-sponsored monograph entitled *Statistical Decision Functions* published the year of his death.^86^

Wald’s productive insight was to conflate statistical decisions— what Wald, following Neyman, labels “the problem of inductive behavior”—as a generalized zero-sum two-person *game* between an “experimenter” and “Nature.”^87^ The *game* is the possible choices available to both experimenter and Nature. For the experimenter: a choice of a “decision function” from all possible decisions and the subsequent decision results (i.e., observations). For Nature: a “choice of [the] true distribution.”^88^ Wald did the difficult mathematical gymnastics of expanding this game to include *infinite* possible choices for both experimenter and Nature, incorporating the use of sequential testing so that after each observation a new decision (e.g., make a classification or take an additional observation) is made in response to the history of previous actions taken by the experimenter.^89^

Here then we can begin to see the analogical and mathematical similarities between Wald’s decision theory and pattern recognition’s OCR problem. In 1957 C. K. Chow, working “in the Electromechanisms Department of the Burroughs Corporation Research Center at Paoli, PA,” suggested decision theory should be used in pattern recognition^90^:

[The optical character] recognition problem is considered to be that of testing multiple hypotheses in the statistical inference. Consequently, the design and evaluation of a recognition system is comparable to a statistical test. Results of decision theory can be applied.^91^

Mindful of the experimental difficulties of comparing error rates of different OCR systems, Chow noted, “Cases may arise where different misrecognitions have different consequences; e.g., the registering of a four as an asterisk may not be as serious an error as registering it as a nine.”^92^ Decision theory offered a ready-made toolkit that could compare engineered pattern recognition systems in more meaningful ways using the concept of a “loss” function:

The criterion of minimum error rate is then no longer appropriate. Instead, the criterion of minimum risk is employed. Proper weights indicate the loss incurred by the system for every possible decision. Proper weights are assigned to measure the consequences of errors, rejections, and correct recognitions. These weights indicate the loss incurred by the system for every possible decision. The loss, which should be regarded as negative utility, may actually represent loss in dollars or unit of utility in measuring the consequence of the system decision.^93^

Chow employed nomenclature already matter-of-factly used in Wald’s 1939 and 1945 papers, including the use of vectors as features typically found in pattern recognition in the 1960s, and was a very early example of Bayesian decision theory employed to a pattern recognition problem. Given “noise statistics, the signal structure, and preassigned weights,” all of which could be determined empirically for a particular OCR system, Chow could not only describe the optimal possible performance for any system according to a particular decision criteria (like the minimization of probability of misclassification), but, more importantly, Chow could describe the ways these different systems would degrade “from characters being corrupted by printing deterioration and/or inherent noise of the [OCR] devices.”^94^

Obtaining empirical probability distributions of actual machines was a formidable challenge to making Chow’s result usable. However, what did facilitate the circulation of Chow’s union of OCR to decision theory was the approach’s agnosticism as to (1) how to choose the features to be examined for a particular pattern recognition problem and (2) how patterns of features might be learned by a particular pattern recognition system. Though beyond the temporal scope of this chapter, agnosticism to these two questions in the 1960s structured the possibility for pattern recognition as a field, and set the stage for pattern recognition’s conflation with machine learning in the 1970s and then subsequent appropriation by artificial intelligence in the 1980s. Today’s machine learning ontology of supervised and unsupervised learning—“learning with a teacher” (using labeled data) and “learning without a teacher” (using unlabeled data), respectively—developed largely in 1960s pattern recognition communities concerned with language and judgement (as already suggested in section “A Decision Procedure When You Don’t Know the Problem Solution Space”), and kept Chow’s agnosticism tied initially to communities familiar with signal processing in engineering applications.^95^

In 1962, following Chow in examining “how to process a set of measurements once that set has been decided upon” and not “what to measure,” N. Abramson, working at Stanford Electronics Laboratory, and D. Braverman, working at Caltech, presented a strategy for “the optimal use of a sequence of prior observations in order to recognize patterns…of a visual, aural, or electromagnetic origin.”^96^ Employing the framing of decision theory, they “show[ed] that the use of prior observations for pattern recognition may be described as a process of learning the statistical characteristics of the patterns involved”—in effect, they presented the paradigm, methods, and notation of *supervised learning*, everything but the word “supervised” itself.^97^ Using supervised learning one could either assume prior probability distributions of the relevant categories and use estimated statistical parameters as the “true” values (i.e., the “parametric” case), or use empirical observations as the “true” values (i.e., the “nonparametric” case).^98^ By 1964, the then-named *supervised* problem was contrasted with the “nonsupervised” case in which the categories are not known beforehand.^99^ If these considerations offered a theoretical framework by which different machines might be usefully compared, this framework was largely ignored by many engineers building prototype pattern recognition systems till the early 1970s. Why?

 The prominence of OCR as an applied problem produced a builder/theorist divide such that “when builders of pattern recognition devices confronted people working on theoretical aspects of pattern classification, especially statistical classification, there was a tendency to question, in the name of practicality and simplicity, the need for theoretical studies in this area.”^100^ If my OCR learning machine identifies the right letter 99% of the time for my data, a pattern-recognition builder might say, why resort to the mathematical sophistry of the theorists?—this attitude was only exacerbated for engineers by the theorists’ “penchant for presenting their work in the unfortunate ‘theorem-proof, theorem-proof’ style” that was endemic to Wald’s work and statistical decision theory generally.^101^ However, ad hoc tinkering and iteration made it difficult for engineers to estimate the challenge of a particular pattern recognition problem except in hindsight. This made it risky—both professionally and financially—to apply pattern recognition to new problems for system builders and their government sponsors; problems anticipated to be solved with ease took years even as other problems thought to be difficult were solved relatively quickly.

Writing in 1968, one researcher summarized the situation: “What is evident is that to a large extent, until very recently, theoretical developments and practical implementation have taken independent paths with little interaction.”^102^ And elsewhere: “we have a jumble of published and unpublished experimental and theoretical results, engineering successes and failures, comments, criticisms and philosophical arguments out of which, hopefully, will emerge some order and maturing of the field.”^103^ Theorizing was a potential means of bringing pattern recognition to bear on new problems by finding ways to categorize the complexity of the particular application and so offer some procedure for estimating the resources required to develop new solutions to new problems and to translate existing solutions into new contexts. But it was also a strategy to unify a hodgepodge of researchers into a *discipline* with its own conferences, revenue streams, professional opportunities, training pipelines, and modes of prestige. By the late 1960s, the desire to know when, why, and how such mechanized classifications-as-judgment would fail “require[d] … a soul-searching methodological examination of the entire enterprise of pattern recognition” that had personal as well as intellectual consequences.^104^

## Epilogues, Epistemic Impotence, and Rearguing the Past

### The Search for Impotence, Mechanized Intuition, & Disciplinary Coherence

Early pattern recognition in the 1950s and 1960s, as articulated by Selfridge and many others, inhabited two registers of discourse. The first register was pedestrian and born of a perceived need for reliability in the face of variegated social data that *could not be anticipated* (i.e., contextual robustness). The second register, in its extreme form, was the very pinnacle of hubris that sought to reproduce the contextual significance of human perception, and even what humans did or could not know, and that made even hyperbolic coeval claims about producing “ intelligence” in artificial intelligence seem levelheaded by comparison.^105^ Both were necessary but not sufficient to argue that pattern recognition was “a vast and explicit endeavor at mechanization of the most fundamental human function of perception and concept formation.”^106^ While the second conference proceedings on the Methodologies of Pattern Recognition in 1969 opened by labeling the field as “one of the most ambitious scientific ventures of this century, requiring collaboration of electronic engineers, physicists, physiologists, psychologists, logicians, mathematicians, and philosophers,” the task of changing pattern recognition “from the status of a programming stunt to … a respectable branch of scientific art” would be achieved, in part, by “explor[ing] the limits beyond which [pattern recognition] will not work.”^107^ Louis Fein’s “Impotence Principles for Machine Intelligence,” the closing paper in the proceedings of first IEEE pattern recognition workshop in 1966, claimed that “the most secure part of our present knowledge is knowing what it is that we cannot know or do,” and cited Godel’s theorems in mathematics, the relativity postulate in relativity, Heisenberg’s uncertainty principle in quantum mechanics, the second law of thermodynamics, and others as examples.^108^ This “important lesson from the history of science,” Fein claimed, had been ignored in “the field of information systems, computers, and automata[.]”^109^ Common to all these impotence principles was a concern for generalized applicability of possible solutions.

The search for generalized rules took many forms, including ways of thematically combining pattern recognition work as a discipline and making this knowledge available to researchers in different disciplines. In the second IEEE conference, one researcher observed the tendency in pattern recognition “towards [...] solutions of very limited problems” and “the scarcity of and means for carrying out comparative evaluations of different approaches,” hindered the dissemination of pattern recognition research, and the “unif[ication] of the field.”^110^ Writing half a century ago, this researcher offered a solution to these challenges remarkly similar in substance to those frequently proffered today: namely, developing a “modus operandi of systems study” for use by “non-specialists” spanning from data set creation to final implementation and its subsequent effects.^111^

Even given the mathematical tools of decision theory numerous researchers noted it was not “adequate for the solution of pattern recognition problems.”^112^ One paper noted decision theory “has had an impact only when it has been supplemented by a substantial amount of heuristic thinking.”^113^ “For the nonformalizable aspects of design,” they concluded, “interactive approaches, namely those in which the human is part of the loop in the design process […] seem to be most promising.”^114^ Another paper noted how pattern recognition involved “inductive ambiguity” in the “act of generating hypotheses” as a result of “extra-evidential factors” that “are entirely invisible,” and that was contingent such that “two hypotheses which are equally well confirmed by the evidence may have different credibilities depending on how well they harmonize with other hypotheses in a theory.”^115^ A 1971 conference paper entitled “A ‘Crisis’ in the Theory of Pattern Recognition,” asked, “what is the novelty, then, that the researchers are after in the theory of pattern recognition?”^116^ No longer, the Russian scientist Alexander Lerner argued, was “learning […] a mysterious phenomenon inherent to living beings”; now it was about minimizing the loss function.^117^ What distinguished pattern recognition from other disciplines? Noting that “physicists are aware that facts alone are not sufficient to select a true theory,” Lerner suggested that picking a “true” theory requires a “selection [that] has been done without any formal rules, by intuition.”^118^ “Computer intuition,” Lerner argued, in pattern recognition was “the introduction of an a priori orderliness [that] enables one to select the best rule from a set of rules that minimize the empirical risk.”^119^

These efforts ultimately highlighted two existential threats to pattern recognition: (1) a confusion among individual researchers about what constituted a good pattern recognition question, and (2) an ambiguity about what distinguished pattern recognition as a distinct field of inquiry (from cybernetics, artificial intelligence, statistics, etc.). Both concerns were manifested through the singular inability of pattern recognition researchers to usefully compare different pattern recognition systems and approaches. By the mid-1970s, *supervised* and *unsupervised* *learning* were established as durable categories that continue to dictate possibility in machine learning to this day, but, as we have seen, were originally developed in the early 1960s. This ontology of learning forged in pattern recognition, and later incorporated into machine learning (the disciplinary formation) *as* machine learning (the set of techniques) did not dispel the practitioners’ sense of the diverse activities that were to constitute the discipline. This was demonstrated in part by a 1973 textbook entitled “Pattern Classification and Scene Analysis,” with chapters on “Bayes Decision Theory,” “ Supervised Learning,” “ Unsupervised Learning,” and “Linear Discriminant Functions,” that began by noting that “this diversity [of the contributions from many different disciplines] also presents serious problems to anyone writing a book on [ pattern recognition].”^120^ They continue:

[Classification theory] provides formal mathematical procedures for classifying patterns once they have been represented abstractly as vectors. Attempts to find domain-independent procedures for constructing these vector representations have not yielded generally useful results. Instead, every problem area has acquired a collection of procedures suited to its special characteristics.^121^

The inability to theorize how to vectorize the world and incorporate these non-necessary “extra-evidential factors,” in hindsight, became precisely one of the key concerns that differentiated pattern recognition as a discipline and shaped the manner in which supervised and unsupervised learning was subsequently valorized and circulated in the late 1970s and early 1980s.

 Pattern recognition did not attain the kind of disciplinary prominence or prestige implied by Watanabe’s claim of “the most ambitious scientific ventures of [the twentieth] century.”^122^ However, the methodologies and epistemological virtues developed in 1950s and 1960s pattern recognition have today become ubiquitous across a dizzying array of disciplines, subjects, and problem domains as machine learning. By the late 1970s, the previous heterogeneity of forms of learning denoted by “machine learning” in the 1950s and 1960s appears to have collapsed into the categories of unsupervised and supervised learning made durable in the 1960s pattern recognition research literature.

### Why the Paucity of Early Machine Learning Histories Has Social and Political Consequences

Histories of machine learning, insofar as they are offered as standalone pieces or as addendums to the history of artificial intelligence, tend to narrate the period from the late mid-1950s to the late 1970s as one in which little progress in machine learning occurred, often using neural network “connectionist” touchstones as a proxy for machine learning progress and foregrounding “AI winters” as a primary driver of machine learning success or failure in both the twentieth and twenty-first centuries. This reflects an instrumental view of the history of science in line with Robert Merton’s sociology of science in which the objective is to help scientists do better science.^123^ Such views of the purpose of the history of science are even shared by the celebrated author of *The Art of Computer Programming* and ACM Turing Award winner Donald Knuth, who has argued that more recent histories of computer science are “dumbed down” in an ill-advised attempt to make it accessible to a reading public beyond computer scientists.^124^ Internalist histories of computer science, Knuth notes, can teach future computer scientists, help them learn from past failures, and develop imagination. For Knuth, the value of these histories should be judged accordingly to how well they succeed at these tasks, but also seems to protect claims of scientific priority and to engage in a certain kind of nostalgia in which prominent scientists become role models. I have no interest in critiquing such uses of history—anymore than I would demand a person read a poem for *only* a particular set of results. Perhaps most readers who have made it this far into the paper will disagree with such a constrained understanding of what history does or should do.^125^

Different histories are written for different purposes, in response to different concerns, and by people with different lived experiences and worldviews. Let Knuth have his technical histories. Let everyone who wants to watch *The Theory of Everything, The Imitation Game,* or *Hidden Figures* as a playbook for a career in cosmology, computing, or aerospace do so. However, historians are painfully aware of how stories we tell about ourselves produce the possibilities for our own action in both subtle and not subtle ways. Our understandings of race, gender, sexuality, and class are profoundly inflected by how we narrate our histories. The U.S. Civil War was about slavery. We shape our world and selves by the stories we tell and the details we choose to make significant. The story of Geoff Hinton unable to find a job because he was working on neural networks isn’t just a story about the state of the field; it’s a subtle commentary by the story teller about the kinds of values or kind of life a researcher *should* have. Einstein invented relativity while working at a patent office. These stories do work for the people who repeat them even as they are also constrained by them. AI winters are the result of researchers overpromising and under delivering rather than a consequence of a Department of Defense shakeup for which artificial intelligence research was an afterthought—*that* story continues to constrain and suggest possibilities for many machine learning researchers today. The development of supervised and unsupervised learning was a particular answer to the problems of knowledge posed within a defuse, transnational network of individuals and institutions across dozens of countries that comprised the pattern recognition community from the 1950s to the 1970s. I sought to recover the epistemic assumptions of this community by tracing the various instantiations of OCR work in local laboratories and in the international technical literature to understand how machine learning came to be seen by some as a compelling method to make social decisions. This led me to a number of new actors who were largely elided in the histories of artificial intelligence, including women and non-white actors who I have cited in this paper that have made important contributions to machine learning and for which further study is required. The present prominence of machine learning in data science and computer science is often narrated in dramatic fashion as a sudden superiority of neural networks over other AI approaches at the end of the twentieth century and the start of the twenty-first. However, as has been shown here, much of the ontology of machine learning and its attendant epistemic justification was set out by the early 1960s. Contemporary debates about the generalizability of machine learning in social decisions rehearses many of the same debates pattern recognition researchers had with each other in the 1960s about how to compare different learning machines using different data sets.

The early 1950s to mid-1970s was an intellectually capacious and generative period for machine learning in the twentieth century—*not* because of any single technical advance but because the research problem of pattern recognition established virtues, practices, and organizing principles for how a useful question was to be identified. Such problem-framing strategies for technical problems circulated across geography and disciplines, in part, because it was also recognized as valuable knowledge by those not doing pattern recognition work. Rather than taking the machine learning terms of supervised and unsupervised learning as self-evident, I see them as research practices in need of explanation, in part, because they inform what constitutes evidence for questions of race, gender, and socioeconomic class now explicitly debated through the implementation of machine learning systems to make important social decisions. The history presented here reflects my questions on the interweaving of technical solutions as political solutions by many contemporary machine learning systems, including Perspective, that are offered as solutions to questions of social order. Narratives pertaining to machine learning are some of the props by which we envision possibility and feasibility. “Now anyone can deploy Google’s troll-fighting AI” said *Wired* when Perspective was publicly released, six months before the discovery of the model’s propensity to label identity words as “toxic” discourse.^126^ Historical narratives and actors who have been underrepresented in the historiography of AI undoubtedly have shaped current day practices. To ignore these historical contingencies in the implementation of machine learning is a shortcoming. Let us be clear-eyed about the amount of work a history of machine learning entails, to what purposes we would put it to, and the generosity of the communities that make this work possible. Let us also be mindful of the challenging situation of early career scholars who recognize that time is not a panacea for justice, and that the insight and intelligence with which we address machine learning systems today will be the linchpin of future bad laws we must later protest.

#### Notes

1. Shapin and Schaffer (1985, p. xlix).
2. Marcus and Davis (2019), Broussard (2018), and Jordan and Mitchell (2015), for instance, situate machine learning as a subfield of artificial intelligence. Valuable historical, sociological, and popular inquiries of artificial intelligence largely distinct from the pattern recognition qua machine learning work discussed in this chapter include Garvey (2019), Dick (2011, 2015), November (2012), Wilson (2010), Nilsson (2010), Boden (1987, 2006), Roland and Shiman (2002), Cordeschi (2002), Forsythe (2001), Crevier (1993), Edwards (1996), Haugeland (1985), Newell (1982), and McCorduck (1979). For operations research, see Thomas (2015), Erikson et al. (2013), and Mirowski (2002). For cybernetics, see Carr (2020), Peters (2016), Kline (2015), Medina (2011), Pickering (2010), and Galison (1994). For cognitive science, see Boden (2006). Valuable historical studies of statistics, risk, and quantification include Dryer (2019), Daston (2018), Igo (2007, 2018), Radin (2017), Bouk (2015), Porter (1986, 1995), Stigler (1986, 1999), Desrosières (1998), Daston (1995), Hacking (1990), and Gigerenzer et al. (1989).
3. Plasek (2016) notes that “the sheer volume and ambit of technical publications produced” indicated a distinct machine learning tradition both simultaneous to and more prolific than early AI research (p. 6). Independently Cardon, Cointet, and Mazières (2018) suggested distinct “connectionist” and “symbolic” approaches using co-citation networks in technical computing literature, noting an excess of connectionist publications over symbolic publications from the 1940s to the mid-1960s. They narrate the second half of the twentieth century and the early twenty-first century as a competition between these two approaches (and their intellectual descendants) and as a series of “AI winters.” Jones (2018) offers a high-level overview of loosely the same period, but focuses on the transnational development of machine learning via debates in exploratory data analysis, pattern recognition, and statistics, especially as these fields changed through interactions between computing practices and modes of epistemic valuing. Nilsson (2010), although ostensibly a monograph of AI histories written by a participant-observer, examines a number of machine learning touchstones (e.g., see chapter 29), and remains valuable for its breadth of scope despite historical idiosyncrasies that reflect Nilsson’s own career. For a technical, participant-observer examination of the touchstones, practices, and narratives of contemporary machine learning researchers, see Mackenzie (2017). For recent explorations of machine learning systems and the contingency in the construction of data sets, see, for instance, Pasquinelli and Joler (2020), Crawford and Paglen (2019), Radin (2017), and many others investigating fairness, accountability, transparency, and ethics of machine learning systems (see note 5).
4. Shapin and Schaffer (1985, p. 332).
5. Regarding computing infrastructures broadly interpreted and their impact on vulnerable populations, see the ACM Conference on Fairness, Accountability, and Transparency (FAccT*). Important references on the subject include Sweeney (2013), O’Neil (2016), Barocas and Selbst (2016), Noble (2018), Eubanks (2018), Buolamwini and Gebru (2018), Ensign, Friedler, Neville, Scheidegger, and Venkatasubramanian (2018), and Benjamin (2019).
6. For the same reasons the usual list of mid-century neural network touchstones often provided as a stand-in for machine learning histories obscures more than it reveals. For common internalist touchstones of connectionist literature, see McCulloch and Pitts (1943), Hebb (1949), Selfridge (1955, 1959), Rosenblatt (1956, 1957), Samuel (1959), and Minsky and Papert (1969), and, when extended into the 1980s to include backwards-propagation, Rumelhart, Hinton, and Williams (1985, 1986a, 1986b), and Hinton (1989). The scientific priority of late twentieth century neural network research remains controversial in internalist debates: see, for instance, Schmidhuber (2015).
7. Daston and Galison (2010, p. 52).
8. Daston and Galison (2010, p. 52).
9. Google (2017a, 2017b). Perspective API was not recommended “as a tool for automated moderation” when released in 2017, but as a tool to assist human online content moderators (Google, 2017b). By 2019 Perspective offered a variety of classifiers, including “severe toxicity,” “identity attack,” “insult,” “profanity,” “threat,” “sexually explicit,” “flirtation,” and a specific *New York Times* classifier (Conversation AI, 2019).
10. Google (2017a); see also Wulczyn, Thain, and Dixon (2017, pp. 1391, 1392–1393); Dixon et al. (2018, p. 68). Each comment was labeled by at least ten workers to ensure uniformity in responses (Wulczyn et al., 2017). Regarding online content moderation and crowdsourced labor, see Gray and Suri (2019) and Gillespie (2018).
11. Marvin (2019).
12. Wulczyn et al. (2017).
13. Wulczyn et al. (2017, pp. 1391, 1394). As of June 4, 2020, an updated version of Google (2017b), lists the “Wikipedia Machine Annotations of Talk Pages” data set as including *all* English Wikipedia talk page comments from 2001–2015, with “approximately 95 million comments.”
14. “I am a gay black woman,” for example, was scored by Perspective as 87% likely to be seen as “toxic” while “I am a man” was 20% (West, 2017).
15. Jigsaw (2018).
16. Dixon et al. (2018, p. 67).
17. Selfridge (1955, pp. 91, 93).
18. Ware (1955, p. 85).
19. “Machine learning” denoted a field of inquiry at least two years prior to the 1956 Dartmouth Artificial Intelligence conference proposal frequently cited for the use of the neologism “artificial intelligence.” See Booth and Booth (1953, p. 211) for a very early use of the term “machine learning” to connote a field of inquiry; see McCarthy, Minsky, Rochester, and Shannon (1955) for a very early use of the term “artificial intelligence.”
20. Newell (1955, pp. 101, 108); see also Ensmenger (2012), and Heath and Allum (1997). Newell identified such “design problems” as computer programming, machine translation, and “abstracting scientific articles” as problems having these characteristics. However, chess became an exemplar for the kind of problem that mid-century logosymbolic AI often studied: research problems in which the logosymbolic rules that dictate possibility could be enumerated in theory even if they could not be exhaustively computed because of limited computing power, time constraints, etc. (Newell, 1955, p. 104). Such learning in the AI context constituted strategies to “extend the set of [rule] expressions” to arrive at a *well*-*defined* answer or final “goal state,” as exemplified by the Logical Theorist, the General Problem Solver, MYCIN, DENDRIL, and others. Newell writes later that learning is “really concerned with generality—with producing a machine general enough to transcend the vision of its designers,” but this vision is specifically one in which “the standard schemes of repetition with the modification of statistical weights is not the most fruitful way to proceed” ( Newell, 1963, p. v). In contrast, learning in mid-century pattern recognition emphasized the variability of “correct” answers.
21. Dinneen (1955, p. 94). Regarding the nature of Selfridge and Dinneen’s collaboration: “Over the past months in a series of after-hour and luncheon meetings, a group of us at the [Lincoln] laboratory have speculated on problems in this area…. Selfridge and I have begun an investigation of one aspect of pattern recognition, that is, the recognition of simple visual patterns” (Dinneen, 1955, p. 94). Dinneen, then a newly minted PhD in 1955 who had just joined Lincoln Labs at MIT in 1954, would go on to become the director of the Lincoln Laboratory at MIT, and later the assistant secretary of defense during the Carter administration.
22. Selfridge (1955, p. 93). Black and white letter images were mapped onto 90 × 90 grids, with black cells having the value 1 and white cells 0 (Dinneen, 1955, p. 94). Two operations were used to produce features: (1) the “averaging function” “eliminate[d] small pieces of noise, isolated 1’s in a field of 0’s” (Selfridge, 1955, p. 92); (2) the “edging operation” “tends to bring out edges and corners […] by evaluating the radial asymmetry around each 1 in the image, and keeping only those 1’s which have more than a certain amount” (Dinneen, 1955, p. 98; Selfridge, 1955, p. 92).
23. This paper concerns itself with equivalence classes used by the historical actors discussed. For an introduction to the complex history of mathematical equivalence, see Asghari (2019).
24. Selfridge (1955, p. 92).
25. Dinneen (1955, p. 94).
26. Dinneen (1955, p. 94).
27. See Note 34.
28. Selfridge (1955, p. 92, emphasis mine).
29. Selfridge (1955, p. 92).
30. Dinneen (1955, p. 94). This system used sequences of averaging and edging operations to produce “blobs” that were subsequently counted to distinguish *A*s and *O*s. Blob-counting, in principle, could not distinguish between *C*s and *U*s (Selfridge, 1955, p. 94), and so serves as an example of a feature that cannot, in principle, distinguish all letter patterns.
31. See “family resemblances” in Wittgenstein (1953, p. 31e).
32. Selfridge (1955, p. 92). Selfridge and Dinneen’s notion of equivalence class is very similar to Pitts and McCulloch’s “equivalent apparitions” that “share a common figure and define a group of transformations that take the equivalents into one another but preserve the figure invariant” (Pitts & McCulloch, 1947, pp. 127–128). Selfridge and Pitts were roommates in graduate school working under Norbert Wiener. Selfridge reports: “The cognition aspect was first sort of tackled by McCulloch and Pitts in their papers in 1943 and 1947. So I talked with Walter [Pitts] a lot about certain things in cognition and the first paper on my work on pattern recognition systems was at the 1955 … conference” (Husbands & Selfridge, 2008, p. 400).
33. Abramson and Braverman (1962, p. S58). Some argued that the first subsidiary problem was a special case of the second: see, for example, Fu (1968a, pp. 2–3).
34. “[T]he whole process of Pattern Recognition is inevitably tied up with ways of determining significance. I suggest—this is my own fancy—that this is the distinction usually made between machines and men…. I do not, however, believe it is a valid distinction” ( Selfridge, 1955, p. 92).
35. Ware (1955, p. 85).
36. Ware (1955, p. 85).
37. Ware (1955, p. 85). Newell articulated this difference in inputs for artificial intelligence and pattern recognition, in part, as “problem-solving versus recognition,” respectively: “Those thinking within the framework of continuous systems concentrated on pattern recognition as the key type of task for machines to do—character recognition, speech recognition, and visual-pattern recognition…. Contrariwise, those thinking within the framework of symbolic systems concentrated on problem-solving as the key type of task for machines to do—game playing, theorem proving, and puzzle solving” ( Newell, 1982, p. 12).
38. Newell (1982, p. 11).
39. In his post hoc history of the mid-1950s split between pattern recognition and artificial intelligence, Newell (1982) notes, that pattern recognition researchers “follow[ed] the lead of physical science and engineering” and employed “differential equations, excitatory and inhibitory networks, statistical and probabilistic systems” (p. 11). “[T]he major historical effect of this [split between symbolic systems and continuous systems] in the sixties,” Newell writes, “was the rather complete separation of those who thought in terms of continuous systems from those who thought in terms of programming systems. The former were the cybernetics and the engineers concerned with pattern recognition. The latter became the AI community. The separation has been strongly institutionalized. The continuous-system fold ended up in electrical engineering departments; the AI folk ended up in computer science departments” ( Newell, 1982, p. 11).
40. A vast literature attests that combinations of mathematical practices, institutional contingencies, labor, cultural values and shared norms, and material computation often inform the ways historical actors understand the world and their relationship to it. For example, Monte Carlo simulations were a way to perform the necessary calculations to build the hydrogen bomb, but came to be seen by some as the correct way of understanding particle physics and so the world (Galison, 1996, 1997). Other examples include proof-proving (Dick, 2011, MacKenzie 2001), precision and the stock market (MacKenzie, 1993, 2008), global climate (Edwards, 2013), computing (Jones, 2016), aerial bombing (Dryer, 2019), scientific atlases (Daston & Galison, 2007), biology (Stevens, 2013), cells (Landecker, 2007), and many, many others.
41. Pickering (2010, p. 20).
42. Pickering (2010, p. 381). Pickering borrows the phrase “exceedingly complex systems” from Stafford Beer. For a study of how cybernetics was employed to make social and political decisions outside of the US context, see, for instance, Medina (2011) and Peters (2016). For cybernetics in the US Cold War context, see Kline (2015).
43. Husbands and Selfridge (2008, p. 398) and Kline (2015, p. 154).
44. See note 21.
45. Lincoln Lab (1955, p. 8). Elided in the historical sources I have located are the Memory Test Computer operators, who were almost certainly women, and whose efforts were likely vital to executing the OCR program. See Dinneen’s acknowledgements in note 55. For discussions of SAGE, see Slayton (2013, chapter 1), Edwards (1996, chapter 3), and Light (2003, chapter 2).
46. This account is documented in Edwards’ *The Closed World* (1996), McCorduck’s *Machines Who Think* (1979), and many others.
47. For explication of trading zones in the history of science, see Galison (2010, 2011); for succinct summary, see Galison (1999).
48. Uhr (1968, p. 159).
49. Fu (1968b, pp. 399–400). The polysemy of learning was not uniformly well-received by pattern recognition researchers: “Through an abuse of the language, the words recognition and learning have been applied to machine systems which implement classification and estimation algorithms” (Kanal, 1968b, p. x).
50. Munson (1969, p. 417).
51. This confusion is due to the appropriation of pattern recognition practices back into the field of artificial intelligence as machine learning in the late 1970s and 1980s. Katz (2017) traces this “malleability of the AI label” today as a “pretext for imposing more metrics upon human endeavors and advancing traditional neoliberal policies” (pp. 1, 2). That may very well be how this confusion has been put to use in the twenty-first century, but the *existence* of this confusion has historical origins that cannot be reduced to claims about discourse, rampant neoliberalism, or corporate takeovers. To do so erases precisely the history I seek to recover.
52. The creation of *precision* in nuclear intercontinental ballistic missiles (MacKenzie, 1993), the application of computer systems to prove proofs and be justified by formal mathematical verification (Dick, 2011, MacKenzie, 2001), and the development of antilock brakes (Johnson, 2009) offer useful parallels for the production of knowledge informed by individual professionalization, local laboratory practices, and transnational knowledge communities.
53. MacKay (1969, p. 409). MacKay was a British physicist, neuroscientist, and early member of the Ratio club.
54. MacKay (1969, p. 409).
55. Dinneen writes in his acknowledgments that “It would have been impossible to obtain the experimental results without the excellent assistance of Miss B. Jensen and Mrs. M. Kannel in programming, coding, and operating the Memory Test Computer. I wish to acknowledge the excellent co-operation of those responsible for the operation of the Memory Test Computer” (Dinneen, 1955, p. 100). The COVID-19 pandemic has made additional archival visits to obtain more information about Jensen and Kannel impossible at the time of writing. For important monographs on gender and labor in the history of mid-twentieth century computing in US and UK contexts, see Ensmenger (2010), Abbate (2012), Hicks (2017), and Rankin (2018).
56. My thinking about the role of disciplinary repertoires in the construction of arguments has benefited and been informed by Slayton (2013).
57. MacKay (1969, p. 410).
58. See, for instance, Brick (1969): “Redundancy, reliability, and uncertainty considerations may make it advantageous to avoid a clear-cut decision, therefore indicating a modus operandi whereby several solutions are encompassed as lower-level processes within a higher-level framework. The ultimate resolution might therefore reside in a meta-structure designed to take advantage of the inherent redundancies” (p. 76).
59. Brick (1969, p. 78).
60. For machine-generated goals in artificial intelligence, see, for instance, Newell (1955, p. 9).
61. Contrast this with Newell’s discussion of the meaning of “ heuristic”: “A ‘heuristic program,’ to be considered successful, must work well on a variety of problems, *and may often be excused if it fails on some.* We often find it worthwhile to introduce a heuristic method which happens to cause occasional failures, if there is an over-all improvement in performance. But imperfect methods are not necessarily heuristic, nor vice versa. Hence ‘ heuristic’ should not be regarded as opposite to ‘foolproof’; this has caused some confusion in the literature” ( Newell, 1955, p. 9, footnote 1, emphasis mine).
62. M. E. Stevens (1961a, p. 333).
63. Rabinow (1968, p. 14).
64. Kanal (1968b, p. xi).
65. The tripartite varieties of “pattern” activity I put forth is informed, in part, by the three-part organization of Kanal (1968a, pp. iii–v) and M. E. Stevens (1961a, p. 333). By 1968, the proceedings of the first workshop on pattern recognition sponsored by the IEEE dedicated the first third explicitly to “character recognition,” and most papers in that volume used OCR—whether hand printed or typed—as the applied example (Kanal, 1968a). If desire and funding to build reading machines originated from 1950s machine translation research, dedicated, in part, to translating Russian science articles into English (Gordon, 2015), the desire for reading machines was sustained by a belief in the late 1950s and early 1960s in the promise of the information sciences to produce “an understanding of [the researcher] himself, how he thinks, how he comes to understand, how he learns, what he wants his goals to be, and why”—technology would change our science and so change ourselves (Swanson, 1966, p. iii). No doubt this situation was made possible in a very direct way by the Department of Defense and National Science Foundation funding made available for “basic research” at universities and companies during the Cold War. For a history of this transition of research from universities to private companies, see Rhode (2013).
66. OCR was not a problem limited to pattern recognition or machine learning. Writing in 1961 for the National Bureau of Standards, Mary Elizabeth Stevens’ extraordinary 1958 survey observes, “‘That the blind may read’ is the earliest recorded objective for research attempts to develop devices capable of reproducing and transcribing printed, typed, or handwritten characters” (M. E. Stevens, 1961b, pp. 2, 152). Likewise, J. Rabinow’s 1968 “State of the Art of Reading Machines” review for the first IEEE Workshop on Pattern Recognition notes, “My own involvement with reading machines came via the route of trying to aid the blind and, particularly, in an attempt to convert an optical image to a tactile one. Even in 1938 this was an old idea and it has been resurrected many times since” (Rabinow, 1968, p. 3, see also pp. 25–26). For the central role of embodiment and disability regarding conceptions of information in cybernetics and communication engineering, see Mills (2011). For the role of reading, disability, and media affordances, see Mills (2012).
67. Watanabe (1969, p. vii).
68. Rabinow (1968, p. 20). Comparing data to an ideal mask, depending on interpretation of features and weights, could be considered a part of the machine learning tradition. However, for the purposes of our discussion, the commercial OCR systems did not “learn” in the senses described in “How Identifying Significance Became a Branch of Engineering” and “Pattern Recognition Eats the World: Learning as a Solution to Technical Problems and to Questions of Social Order”.
69. Rabinow (1968, pp. 20–21).
70. In 1958 Selfridge presented a pattern recognition strategy capable of learning letter features in a system he called “pandemonium,” in an explicit reference to Milton’s *Paradise Lost* (see Selfridge, 1959), but, despite pandemonium’s prominence in some historical accounts, this system was not widely imitated.
71. Rabinow (1968, pp. 23–24).
72. Groner (1968, p. 103).
73. Bernstein (1968).
74. Munson (1968, pp. 126–127).
75. Munson (1968, pp. 129, 130).
76. Munson (1968, p. 136).
77. Munson (1968, p. 137).
78. Munson (1968, p. 139).
79. See the *U*s and *C*s example in note 30.
80. Rabinow (1968, pp. 27–28) and Hessinger (1968).
81. For these last 4 examples, see Sheinberg (1968, pp. 38–39).
82. See, for instance, Chow (1957, p. 252) and Brick (1969, p. 76).
83. Wald (1950, pp. 21–22, 10, 28–29). For a discussion of Wald’s statistical “general [decision] problem” in relationship to Neyman-Pearson hypothesis testing, see especially Wald (1939, p. 300). For an internalist historical account of the mathematics of decision theory, see Wald (1950, pp. 28–31). For an example of Wald’s work on quality control in US factories, see Columbia Statistical Research Group (1945). For a broader historical account of decision theory as it pertained to operations research, linear programming, and systems design in the US and UK contexts, see Thomas (2015). For the interwoven transnational application of the decision theory in rural Indian reconstruction and US military contexts, especially constructions of certainty in political discourse via “confidence interval logics” and “deconstruction data” sites, see Dryer (2019), especially Chapter 10.1007/978-3-030-56286-1_4. For the debates about Cold War “rationality,” of which Wald’s decision theory is an important rhetorical and technical tool for debate, see Erikson et al. (2013).
84. Weiss (1992, pp. 335–336).
85. Wald’s 1939 paper was cited as his most important contribution by his friend and colleague Jacob Wolfowitz (Wald, 1939; Wolfowitz, 1952). In this paper Wald articulated a very early, very generalized version of statistical decision theory, while also introducing loss functions, Bayes decision solutions, the minimax procedure, and more that comprises elementary textbook machine learning today. Wald’s sequential analysis work was classified in 1943, but later published in Wald (1945). See “historical note” in Wald (1945, pp. 119–122).
86. Wald (1950); see also Wald (1949).
87. Wald (1950, pp. 24, 26–27).
88. Wald (1950, p. 27).
89. Weiss (1992) notes: “The Bayesian approach to statistical problems is at least partly a reaction to minimax decision rules…. [M]inimax decision rules are often very conservative. This was inevitable, considering the fact that they come from the theory of zero-sum two-person games, in which players are forced to be antagonistic. If the statistician is playing against Nature, the question is whether nature is that malevolent. A Bayesian feels that he knows what a priori distribution is being used by nature” (p. 340). Wald (1950) notes: “Whereas the experimenter wishes to maximize the risk … we can hardly say that Nature wishes to maximize [risk]. Nevertheless, since Nature’s choice is unknown to the experimenter, it is perhaps not unreasonable for the experimenter to behave as if Nature wanted to maximize the risk” (p. 27).
90. Chow (1957, p. 254).
91. Chow (1957, p. 249, see also p. 248).
92. Chow (1957, p. 249).
93. Chow (1957, p. 249).
94. Chow (1957, p. 247).
95. The phrases and metaphors of *learning with/without a teacher* have a long history. For early examples, see Turing (1948) and Booth and Booth (1953). In the explicit context of supervised and unsupervised learning, see Cooper (1969, p. 99).
96. Abramson and Braverman (1962, p. S58).
97. Abramson and Braverman (1962, p. S58).
98. For the historical source providing this explanation, see Cooper (1969, p. 99).
99. Cooper (1969).
100. Kanal (1968b, p. ix).
101. Kanal, (1968b, p. x). See also Kanal and Chandrasekaran (1969, 324).
102. Kanal (1968b, p. ix). Kanal notes that “most of the commercially available optical character readers have used heuristic techniques almost exclusively. Part of the reason for this is that, often, commercial readers have been developed by hardware oriented engineers not familiar with or convinced about statistical classification theory” (p. ix).
103. Kanal (1968b, p. vii).
104. Watanabe (1969, p. vii).
105. For an elegant articulation of both “registers,” see, for instance, Kanal and Chandrasekaran (1969, pp. 317–318).
106. Watanabe (1969, p. vii).
107. Watanabe (1969, p. vii).
108. Fein (1968, p. 443).
109. Fein (1968, p. 445).
110. Brick (1969, pp. 75, 83). For a detailed list of “feature extraction” techniques, “ classification and recognition (decision) techniques” and much more, see pp. 89–96.
111. Brick (1969, pp. 78, 80).
112. Kanal and Chandrasekaran (1969, p. 324).
113. Kanal and Chandrasekaran (1969, p. 324).
114. Kanal and Chandrasekaran (1969, p. 331).
115. Watanabe (1969, pp. 521–522, 525).
116. Lerner (1972, p. 368).
117. Lerner (1972, p. 368).
118. Lerner (1972, p. 369).
119. Lerner (1972, p. 371).
120. Duda and Hart (1973, p. vii).
121. Duda and Hart (1973, p. vii).
122. Watanbe (1969, p. vii).
123. See Zuckerman (1988) for a discussion of Merton’s sociological project.
124. See Haigh (2015) for discussion and references to Knuth’s lecture entitled “Let’s Not Dumb Down the History of Computer Science.”
125. For an introduction to history of such internalist and externalist debates in the history of science and science and technology studies, see, for instance, Daston (2009).
126. Greenburg (2017).
